# Compensation of Missing Wedge Effects with Sequential Statistical Reconstruction in Electron Tomography

**DOI:** 10.1371/journal.pone.0108978

**Published:** 2014-10-03

**Authors:** Lassi Paavolainen, Erman Acar, Uygar Tuna, Sari Peltonen, Toshio Moriya, Pan Soonsawad, Varpu Marjomäki, R. Holland Cheng, Ulla Ruotsalainen

**Affiliations:** 1 Department of Biological and Environmental Science/Nanoscience Center, University of Jyväskylä, Jyväskylä, Finland; 2 Department of Signal Processing, Tampere University of Technology, Tampere, Finland; 3 BioMediTech, Tampere University of Technology, Tampere, Finland; 4 Department of Molecular and Cellular Biology, University of California Davis, Davis, California, United States of America; 5 Department of Anatomy, Faculty of Dentistry, Mahidol University, Bangkok, Thailand; UMR Inserm U1052/CNRS 5286, France

## Abstract

Electron tomography (ET) of biological samples is used to study the organization and the structure of the whole cell and subcellular complexes in great detail. However, projections cannot be acquired over full tilt angle range with biological samples in electron microscopy. ET image reconstruction can be considered an ill-posed problem because of this missing information. This results in artifacts, seen as the loss of three-dimensional (3D) resolution in the reconstructed images. The goal of this study was to achieve isotropic resolution with a statistical reconstruction method, sequential maximum a posteriori expectation maximization (sMAP-EM), using no prior morphological knowledge about the specimen. The missing wedge effects on sMAP-EM were examined with a synthetic cell phantom to assess the effects of noise. An experimental dataset of a multivesicular body was evaluated with a number of gold particles. An ellipsoid fitting based method was developed to realize the quantitative measures elongation and contrast in an automated, objective, and reliable way. The method statistically evaluates the sub-volumes containing gold particles randomly located in various parts of the whole volume, thus giving information about the robustness of the volume reconstruction. The quantitative results were also compared with reconstructions made with widely-used weighted backprojection and simultaneous iterative reconstruction technique methods. The results showed that the proposed sMAP-EM method significantly suppresses the effects of the missing information producing isotropic resolution. Furthermore, this method improves the contrast ratio, enhancing the applicability of further automatic and semi-automatic analysis. These improvements in ET reconstruction by sMAP-EM enable analysis of subcellular structures with higher three-dimensional resolution and contrast than conventional methods.

## Introduction

Electron tomography (ET) of cellular samples is a widely used technique for three-dimensional (3D) reconstruction of complex subcellular structures at a resolution enabling the detection of macromolecular complex organizations [1]. Generally, in tomography a 3D model of an object is reconstructed from a collection of two-dimensional (2D) projection images of the sample taken in multiple orientations. In its simplest form, either the sample or the radiation source and detector are rotated around a single axis for full 180 or 360 degrees with fixed intervals (typically 1–2°) while projection images are taken [1]. ET is a combination of this computed tomography and electron microscopy, and fills the resolution gap between the structural methods at the sub-nanometer level, such as single-particle reconstruction, and those at the sub-micrometer level using optical microscopy. In ET, transmission electron microscope (TEM) is used to image typically 200–500nm thick samples [2] eliminating the need for finer sectioning to visualize the sample volume. Using modern sample preparation techniques, like cryo-electron microscopy methods, ET enables studying the physiological mechanisms of subcellular organelles in their native context [1], [2].

To understand the highly complex mechanisms of cell activities, like the signal pathways and mechanisms of virus infections, it is crucial to study various aspects of the cell such as the morphological abnormalities of the whole cell, organelles, or intracellular compartment membranes, and localization or distribution changes of related proteins. These phenomena occur in 3D space. However, while the TEM imaging allows a high resolution observation, the images lose the information of the density distribution along the z-direction (direction of the electron beam, depth), occluding the fine features and distance between two objects along this direction. Therefore, the 3D reconstructions of the target objects using ET have been important for these biological studies. However, ET of biological samples holds two major limitations in image acquisition of tilt series for tomography: a limited range of observable tilt angles and an extremely low signal-to-noise ratio (SNR). In the general case, the sample cannot be imaged in full 180° tilt angle range since the structure of the sample holder and limited space between the pole pieces of the objective lens prevent acquiring images with high tilt angles [3]. This missing angular range is known as *missing wedge*. In addition, at 60° tilt angle, the electron beam has to pass approximately twice as much material of the slab-shaped sample as at zero degree [4]. This makes the quality of high tilt angle images worse. Typically, ±60°–70° tilt angle range is used in ET. Together, these technological challenges cause anisotropic resolution seen as elongation and blurring of the objects in the z-direction. In order to reduce the missing information and minimize these artifacts, additional data is acquired from different tilt axes (double tilt axis [5], [6], conical tilt axis [7]). The missing wedge is reduced to a missing pyramid with double tilt axis, and a missing cone with conical tilt axis by increasing the information coverage in the Fourier domain. Nevertheless, the usage of these techniques has not been as widespread as single axis tilting because they are technically demanding. They require a more complex sample holder and the alignment of the projections is more complicated in comparison with the single axis tilting geometry. Moreover, the total dose of electrons received by the specimen increases by twice with the double tilt geometry. An excess of electron dose damages target objects in a sample by destroying the smallest details, and can induce deformation such as shrinkage in the beam direction [4]. To avoid possible damages to objects, it is important to keep the cumulative electron dosage as low as possible. However, the low electron dosage decreases SNR, and as such, also detectable resolution. A compromise between the number of projections and the used dosage on each projection needs to be found to maximize the resolution of the imaged object.

The most used image reconstruction methods in ET are weighted backprojection (WBP) [8], [9] and simultaneous iterative reconstruction technique (SIRT) [10], due to their simplicity and wide availability in tomographic reconstruction software packages. However, WBP is sensitive to the missing wedge and can result in severe artifacts in limited angle tomography such as ET [11]. SIRT is an iterative method that approximates 3D volume by minimizing the difference between the original projections and projections of the reconstructed volume. It was developed to be usable also in ET with large missing wedge using a sparse set of projections. However, it does not fill the missing wedge and is not able to handle varying sample depth between low and high tilt angles [12]. Recently, a SIRT method using WBP in initialization and backprojection steps was presented [13] to improve reconstruction compared with either of the methods separately.

Regularization and *a priori* knowledge have been used to reduce artifacts, which are common in traditional reconstruction methods used for ET. Angle dependent non-linear anisotropic diffusion filtering [14] has been applied to projection images to compensate the varying depth of the sample. To compensate the missing wedge, total variation and directional smoothing [15] was applied to projection images. Shape-based regularization using prior models of particles included in the sample [16] and segmented mask as a prior in modified SIRT [17] were presented recently. However, in general ET reconstruction problem, no *a priori* knowledge of the objects in the sample is available, unless external objects, such as colloidal gold particles, are added into the sample. Recently, compressed sensing was applied to ET reconstruction to reduce artifacts caused by the missing wedge [18], [19]. Compressed sensing reconstruction methods require *a priori* knowledge of the sparse representation of the objects in the sample. This representation was provided by the image gradient, improving especially boundary regions in the reconstructions.

The goal of this study was to gain isotropic resolution for which we applied a new statistical image reconstruction method in ET, capable of correcting the effects of the missing wedge even when *a priori* knowledge of the objects is not available. Statistical methods have been used in e.g. emission tomography (PET, SPECT) to successfully reconstruct 3D images from noisy projection data [20]–[22]. For this study, we chose the sequential maximum a posteriori expectation maximization (sMAP-EM) method [20], which searches for the most likely cross-sectional images given the measured projections from TEM. We regularized the iterative reconstruction with a median filtered image of the previous iteration in a weighted one-step late algorithm to control the noise in the reconstructed images [23]. The reconstruction was expected to estimate values for the missing wedge during the iterations and to form a 3D image from the measurements. Instead of running the iterative reconstruction once to convergence with a chosen weight for the regularization, we applied the method sequentially. In these sequences, the previous result was initializing the successive one while the weight of the regularization was gradually decreased sequence by sequence. By this way, sMAP-EM improves the image quality step by step during the sequences.

To evaluate sMAP-EM reconstruction method, we utilized synthetic cell datasets and an experimental data consisting of several gold particles as test targets. Gold particles with known shape and size are often used as reference objects in biological samples. An automatic ellipsoid fitting based method, utilizing this knowledge about the gold particles, was developed in this study to measure elongation and contrast in the reconstructions. We compared sMAP-EM reconstructions with the WBP and SIRT results. All the qualitative and quantitative evaluations showed that the sMAP-EM image reconstruction method gives the best isotropic results and the best contrast in the images.

## Materials and Methods

### Datasets

#### Cell phantoms

**Figure 1:**
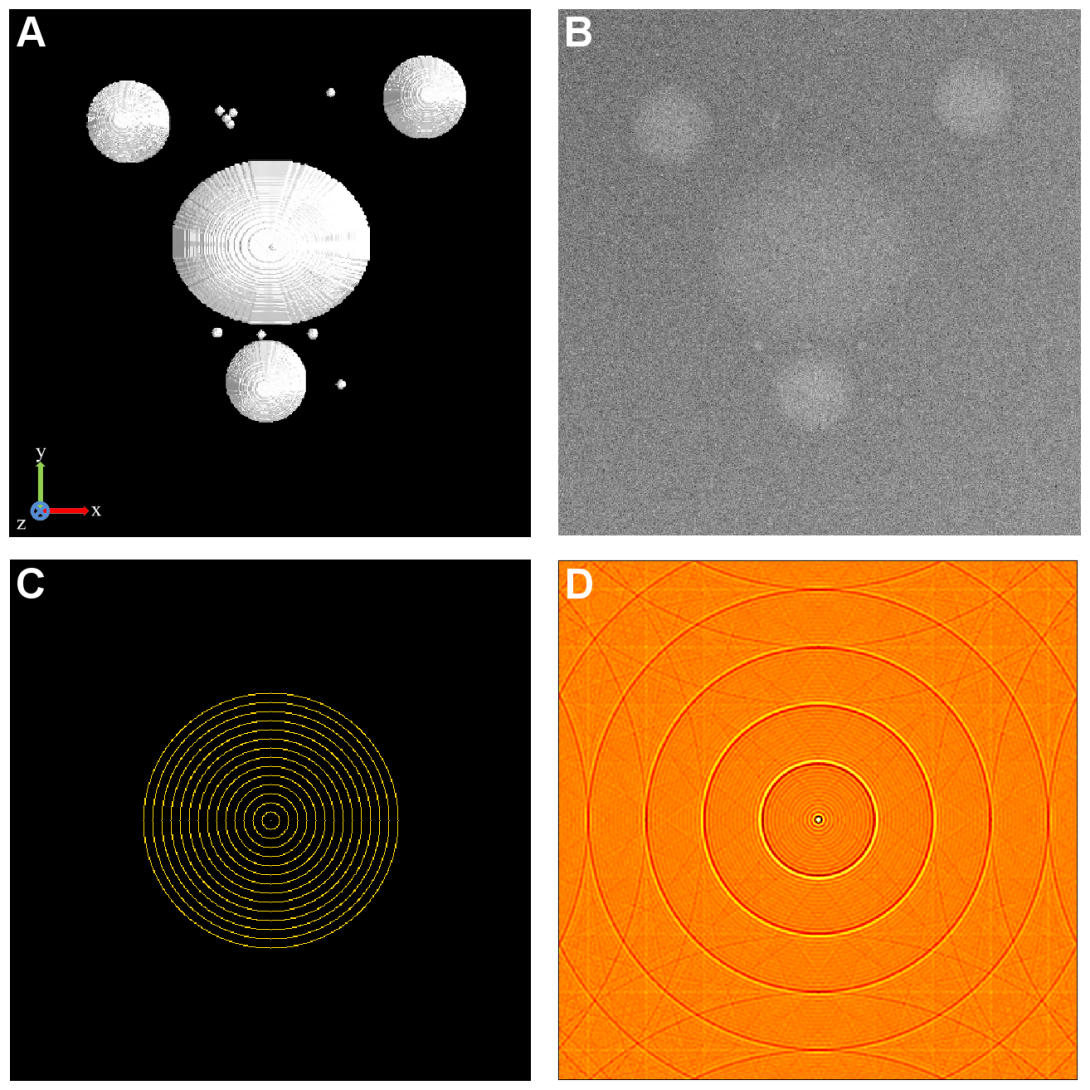
Phantom datasets. Synthetic datasets generated in the study for quantitative analysis with known truth. A) Surface rendering of the cell phantom dataset. B) Zero tilt projection of the cell phantom with 16.1% noise contamination according to the coefficient-of-variation test. C) The artificial pattern synthesized to observe missing wedge effects in the frequency domain. D) The spatial domain image corresponding to the artificial pattern.

The cell phantom (Figure 1A,B) was created in order to test the developed method with a realistic numerical phantom of an intracellular structure. The constructed phantom is of the size 512×512×128 in the (x, y, z) directions, with isotropic voxel size of 1 nm. x, y and z axes are defined as the horizontal axis of the projection images, the tilt axis, and the direction of the electron beam, respectively. It contains 3 large spherical objects mimicking virus particles. Each virus particle has a diameter of 80nm. A large ellipsoidal object representing a cell vesicle was also included. This vesicle was cut in half from the middle z-section emulating the microtomy process. The lengths of the axes of this vesicle are 102, 85 and 51nm. Virus particles and vesicle were created using textures from the reconstruction of experimental data. Additionally, 11 small and dense spherical objects presenting gold particles were added. These gold particles, having diameter 7, 9 and 11nm, had high density to simulate TEM imaging. Non-uniform background density was set to smoothly vary 2.4% between the minimum and maximum density. Virtual projections were calculated with 1° increment using four different tilt angle ranges (±40°, ±50°, ±60°, ±70°) to vary the size of the missing wedge. With the addition of noise to the projection data, two main components of noise in experimental TEM projections were simulated; Poisson noise due to electron counting and Gaussian noise due to the CCD sensor. The obtained projections were contaminated with two different levels corresponding to approximately 16.1% (noise level 1, NL 1, Figure 1B) and 18.0% (noise level 2, NL 2) noise contamination according to the coefficient-of-variation test.

#### Synthetic pattern for frequency domain analysis for missing wedge area

The analysis was done to study what is produced in the region of missing wedge area by the reconstruction methods and how it evolves with changing tilt angle. For this analysis, first, a well-defined frequency pattern (Figure 1C) was synthesized to better observe how the missing wedge is filled. Then the artificial image (Figure 1D) corresponding to this pattern was obtained with inverse 2D fast Fourier transformation. The amplitudes of the frequencies were adjusted so that there were no pixels with negative values in the spatial domain image. Finally, virtual projections of this image were calculated with 1° increment for ±60° tilt angle range. These projections were reconstructed with all three reconstruction methods. Only for sMAP-EM, projections with the 10° increment step of the wedge size were reconstructed for finer observation (Figure S3).

#### Experimental vesicle data

**Figure 2:**
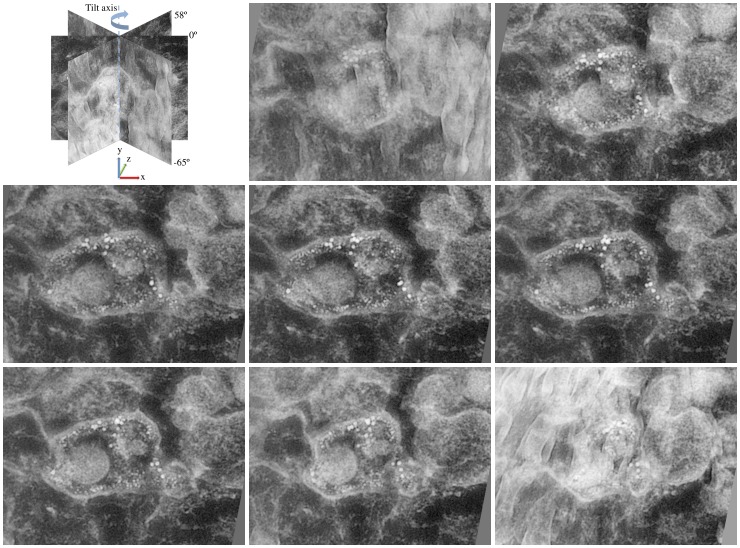
Experimental vesicle data. Altogether 124 projections of the experimental data were taken approximately from −65° to 58° tilt angle range with 1° increments. The experimental data includes a multivesicular body, intraluminal vesicles and gold particles of two diameters. Projection tilt angles from left to right, and from top to bottom: −64.97°, −46.93°, −29.00°, −11.97°, 6.16°, 24.04°, 41.07°, 58.05°. Projections are presented with inverted intensities to improve the visualization.

An experimental sample of a multivesicular body (MVB) (Figure 2) was prepared by high-pressure freezing and freeze substitution as in [24]. The sample was used to study breakages in the membranes of virus-induced MVB and intraluminal vesicles suggesting genome release to the cytoplasm. Also, two specific targets, echovirus 1 and *α*2*β*1-integrin receptor, were labeled with gold particles of 14nm and 6 nm of size, respectively, to study the localization and distribution of these targets in the virus-induced MVB. A series of single axis TEM images were obtained with the JEM-2100F Field Emission Electron Microscope (JEOL Ltd., Tokyo, Japan) at a voltage of 200 kV. It comprises 124 images of the MVB taken at an interval of approximately 1° in the angular range [−65°, +58°], with the magnification of 10k. Before reconstruction, the projection images were aligned by cross-correlation using the 14nm gold particles as markers. The dimensions of the original projection images were 4096×4096 which were cropped to size 958×712 pixels used for reconstruction. The pixel size was 1nm.

The projection data and reconstructions of all datasets are available at our supplementary data site: http://pioms.ucdavis.edu/pone/smap-em.

### Sequential MAP-EM Image Reconstruction Method

**Figure 3:**
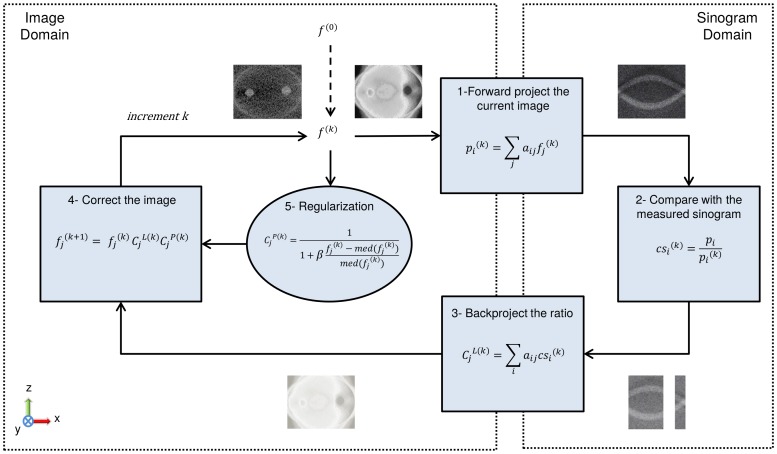
One sequence of the sMAP-EM reconstruction method for a single slice of the sample volume. *f* ^(0)^ is the initial image, 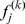 is the reconstruction result of the *j^th^* pixel at *k^th^* iteration, *a_ij_* is the system matrix element defining the contribution of the *j^th^* pixel to the *i^th^* projection, *p_i_* is the *i^th^* projection, *med*(⋅) is the median filter with a 3×3 kernel size, and *β* is the predefined sequence dependent coefficient defining the amount of regularization. The blocks (1), (2), and (3) yield the ML-EM correction factor, 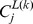, and the block (5) yields the a priori correction factor 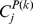. Two correction factors are combined in the block (4) to update the current estimate.

**Figure 4:**
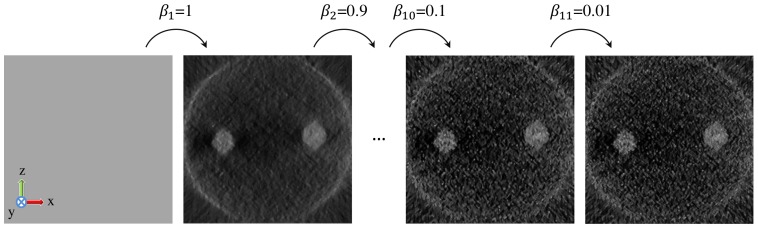
Sequential adaptation of the regularization weight, *β*. The sequences are initialized with an image of ones (the leftmost image). At the end of sequences with decreased *β* values, the final reconstruction image is obtained (the rightmost image). In total, 1000 iterations are performed in 11 sequences.

Maximum likelihood (ML) estimation was used previously for tomographic reconstruction considering the Poisson nature of the measured data [25]. The method searches for the image maximizing the likelihood of the measured data to occur (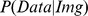) using the iterative algorithm, expectation maximization (EM). In each ML-EM iteration, the estimated image is projected to the sinogram domain and compared with the measured sinogram proportionally. The result of this comparison is back-projected to the image domain to obtain a correction image which is used to update the current image estimate multiplicatively. The method produces statistically unbiased but noisy images [26]. In order to improve the image quality, a priori probability distribution was used [27]–[29] based on the Bayes' rule:
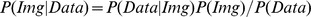
where 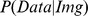 is the likelihood function, *P*(*Img*) and *P*(*Data*) are the a priori probability distributions. 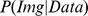 is the a posteriori probability distribution function to be maximized with EM algorithm in maximum a posteriori expectation maximization (MAP-EM) approaches [30]. Since *P*(*Data*) is constant, it does not have influence on the solution. For *P*(*Img*), Alenius et al. [23] suggested median root prior, based on the assumption that the intensity values are similar in the small neighborhood of each pixel of the reconstructed image. The advantage of median filtering is that it is good at preserving edges while reducing the noise. In our study, a sequential version of this median root prior based MAP-EM approach [20] was used for solving the missing wedge problem. One sequence of the method is illustrated for a single slice in Figure 3. The blocks (1), (2), and (3) represent the ML-EM approach and the block (5) represents a priori information introduced. In the block (4) the result of ML-EM and a priori information are combined together to update the current estimate. The iterations continue with a constant predefined regularization weight *β*, in each sequence. The value of *β* is varied throughout the sequences as shown in Figure 4.

The first sequence was initialized with an image of a non-zero value and the value of *β* was set to 1 which corresponds to full regularization. The successive sequences were initialized with the resulting image of the previous one and the *β* value was decreased gradually sequence by sequence. It was reduced to 0.1 linearly in 10 steps and the final sequence was performed with *β = *0.01. By decreasing the *β* value, the blurring effect of the regularization filter was reduced. Therefore the resolution and the intensity contrast were enhanced. However, *β* was never set to 0 in order to avoid checkerboard artifacts observed when a high number of iterations is used without regularization [31]. The number of iterations in each sequence of sMAP-EM was chosen to be sufficiently large to satisfy convergence for reconstructions at different noise levels and different samples.

**Figure 5:**
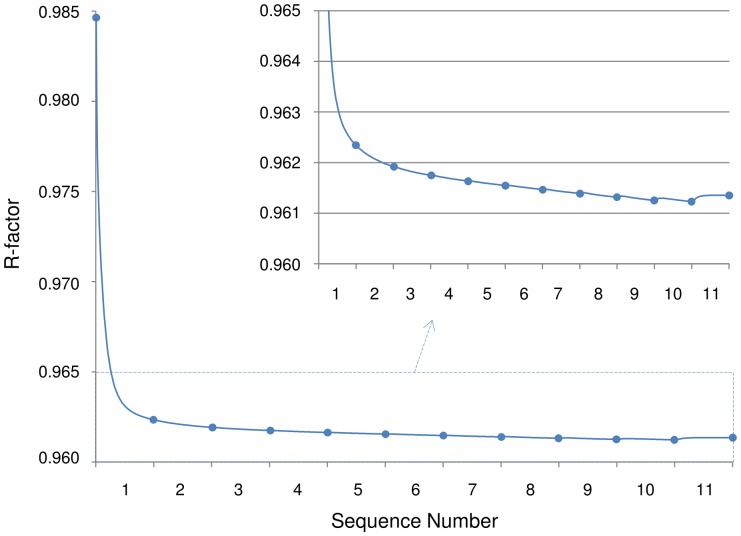
Illustration of the change of the R-factor throughout the iterations. R-factor decreases smoothly during the first 10 sequences. At the final sequence, a small increase is observed due to the noise contamination while enhancing the resolution and the contrast.

To illustrate the change in the reconstructed image during sequences, the R-factor was calculated using a small region of interest (ROI) including a virus particle in one slice of the cell phantom (NL1) data as:
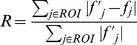
where *f_j_* is the reconstruction result and 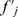 is the ground truth of the *j^th^* pixel. The change in the R-factor throughout the sequences is shown in Figure 5. For the first 10 sequences, R-factor decreases smoothly during the iterations. At the final sequence, the decrease of *β* value from 0.1 to 0.01 introduces some noise to the image which yields a small increase in the R-factor curve. However, by decreasing the weight of the low pass regularization filter, the visual quality of the image is enhanced at the final sequence in terms of the resolution and the contrast.

The reconstruction method was implemented in MATLAB (MathWorks Inc., MA, USA) and the code (available at: https://www.cs.tut.fi/sgn/m2obsi/m2obsiWWW/demos/mapem/MAPEM.html) was run on the computer grid Techila (Techila Technologies Ltd., Tampere, Finland). The sample volume was divided into slices perpendicular to the tilt axis and sMAP-EM was applied to the image of each slice in parallel. The resulting 3D volume was obtained by concatenating the reconstructed 2D images. In CPU time, it took approximately 960 hours for the cell phantom dataset and 4230 hours for the experimental vesicle dataset to obtain the reconstruction results. Approximately 120 AMD 64-bit processor worker computers participated in the experiments. Therefore the reconstruction of the cell phantom data took about 8 hours and the experimental vesicle data about 35 hours.

### Conventional reconstruction methods for comparison: WBP & SIRT

Widely used implementations of WBP and SIRT available in Tomo3D software [32] were used to test the methods. The WBP method simply distributes the projection data over the image plane proportionally to the contribution of each image element (pixel). The WBP method can be mathematically expressed as
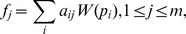
where *f_j_* is the reconstruction result of the *j^th^* pixel, *a_ij_* is the system matrix element defining the contribution of the *j^th^* pixel to the *i^th^* projection, *p_i_* is the *i^th^* projection, *m* is the total number of pixels, and *W*(⋅) represents the filtering operation. In this study, a ramp filter and a Hamming filter with cutoff frequency 0.5 cycles/pixel were used in WBP reconstructions. Ramp filter was used to compensate the blurring artifact resulting from simple backprojection and the Hamming filter was used to suppress the high frequency noise amplified by the ramp filter.

SIRT is another well-known reconstruction method which enhances the image iteratively using forward and backward projections. The method is initialized with an arbitrary reconstruction estimate. First, the image is projected to the sinogram domain. Next, the difference between the measured projections and the projections from the current reconstruction is calculated. Finally, the difference is back-projected to the image domain to update the current image estimate. The method can be formulated as [10]:
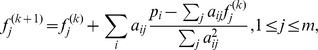
where 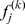 is the reconstruction result of the *j^th^* pixel at the *k^th^* iteration. SIRT with long object compensation was used in this study as proposed by [33]. The method is weighting the projections considering the length of the parallel rays clipped to the bounding box of the reconstruction region to eliminate the artifacts at the boundary of the reconstructed ROI. SIRT was run with 30 iterations in cell phantom data and frequency domain analysis, while 50 iterations were used in experimental vesicle data to improve visual quality. Both iteration numbers are appropriate according to the evaluations made in [33].

### Ellipsoid fitting based evaluation methods

Both visual and quantitative evaluations were made from all reconstructions. An objective ellipsoid fitting based method was developed in BioImageXD software [34] to quantitatively measure accuracy and contrast using gold particles in the reconstructed volume. The method generates a set of ellipsoids *x* ^2^/*a* ^2^+*y* ^2^/*b* ^2^+*z* ^2^/*c* ^2^ = 1 with varying ellipsoidal parameters 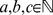. Voxels inside the ellipsoids are set as a mask. Normalized cross-correlation is used to find the location of gold particle in the reconstruction from a small volume around the initial location. Parameters of the best fitting ellipsoid for each gold particle are extracted from correlations by selecting the highest correlation coefficient.

In general, low SNR decreases the accuracy of object detection in images. Low SNR also affects the ability of the ellipsoid fitting based method to find a clear gold particle boundary. This applies especially for the WBP and SIRT reconstructions, whereas sMAP-EM reconstructions were quantified accurately even with the highest level of noise used. The accuracy of ellipsoid fitting based method was tested in all reconstruction methods using projection images with varying levels of Gaussian noise. The method gives reliable results (see Figure S1) for sMAP-EM with all noise levels. The results are also stable for projections having NL1 or higher SNR, but less reliable for NL2 with WBP and SIRT methods. It can be expected that the ellipsoid fitting method does not give reliable results for the WBP and SIRT reconstructions from projections with lower SNR than in NL2 images. However, the ellipsoid fitting based method can be used to compare different reconstruction methods and data acquisition procedures in an automated, objective, and reliable way under certain amount of noise.

To assess the accuracy in 3D, ground-truth gold particles in the cell phantom data were analyzed. Parameters of fitted ellipsoids were compared with the radius (*r*) of ground-truth gold particles in the original volume. The fitted ellipsoid axis length absolute error in all directions was calculated as 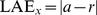, 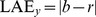 and 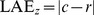.

In order to measure the effect of the missing wedge on the resolution, elongations in all axes were analyzed from the reconstructions. With isotropic resolution, elongation would be 1.0 in all directions, as gold particles are spherical in the nanometer scale. However, as a result of the missing wedge, the images of the gold particles tend to be elongated especially in the z-direction. Elongations were calculated directly from the fitted ellipsoid parameters in all directions as *e_yx_* = *b*/*a*, *e_zx_* = *c*/*a* and *e_zy_* = *c*/*b*.

The contrast in the reconstructions was evaluated using contrast ratio (CR) to quantify how well gold particles can be visualized and analyzed. The higher CR values mean higher contrast, and better visibility, in the reconstructed volumes. CR was calculated as
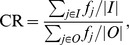
where *f* is the reconstructed volume, 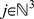 is the voxel index, *f_j_* is the value of voxel *j* in the volume *f*, and the sets *I* and *O* are the voxels inside and outside the volume of interest (VOI) defined by the fitted ellipsoid, respectively. The voxels outside the VOI were defined to construct a volume around the VOI with the same shape but twice the volume of the VOI.

## Results

### Synthetic data reconstructions

**Figure 6:**
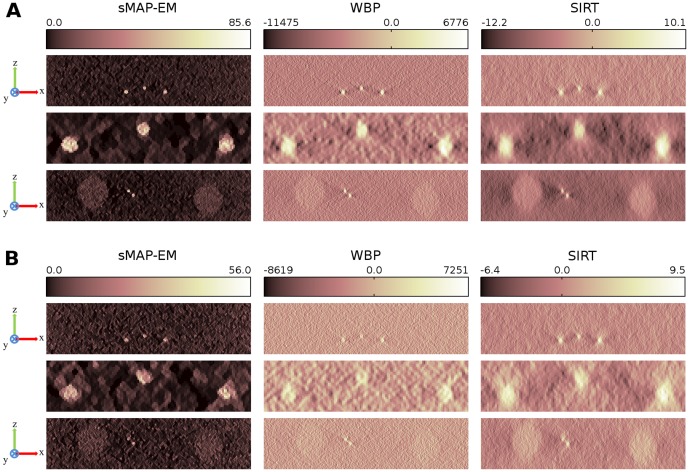
Orthogonal x–z slices from the cell phantom reconstructions. Orthogonal x–z slices from the reconstructions with projections in ±60° tilt angle range having (A) 16.1% (noise level 1, NL1) and (B) 18.0% (noise level 2, NL2) noise contamination according to the coefficient-of-variation test. Slices show gold particles (top row) and virus particles (bottom row). The middle row images present zoomed region of the gold particles in the top row images. The full dynamic range of raw pixel values is presented with pseudo-color. The artifacts caused by missing wedge are clearly present in the WBP and SIRT reconstructions from NL1 projections, whereas sMAP-EM is able to compensate the missing information better. The reconstructions from NL2 projections have lower contrast and as such, also the artifacts are less visible.

To observe the effect of noise to the reconstructions, we reconstructed the cell phantom data from two projection noise levels. Figure 6 shows different slices from the reconstructed volumes. sMAP-EM reconstruction shows clearly better contrast in the images. All methods produce results where objects are detectable with lower noise. However, reconstructions of higher noise level are more different. The used SIRT reconstruction with long object compensation clearly includes more smoothing of the data. The WBP and the SIRT reconstructions showed shadows near the objects in the lateral (x–y) plane and clear artifacts in x-z slice due to the missing wedge. The artifacts were more visible in the reconstructions at the lower noise level for the WBP and SIRT methods.

**Figure 7:**
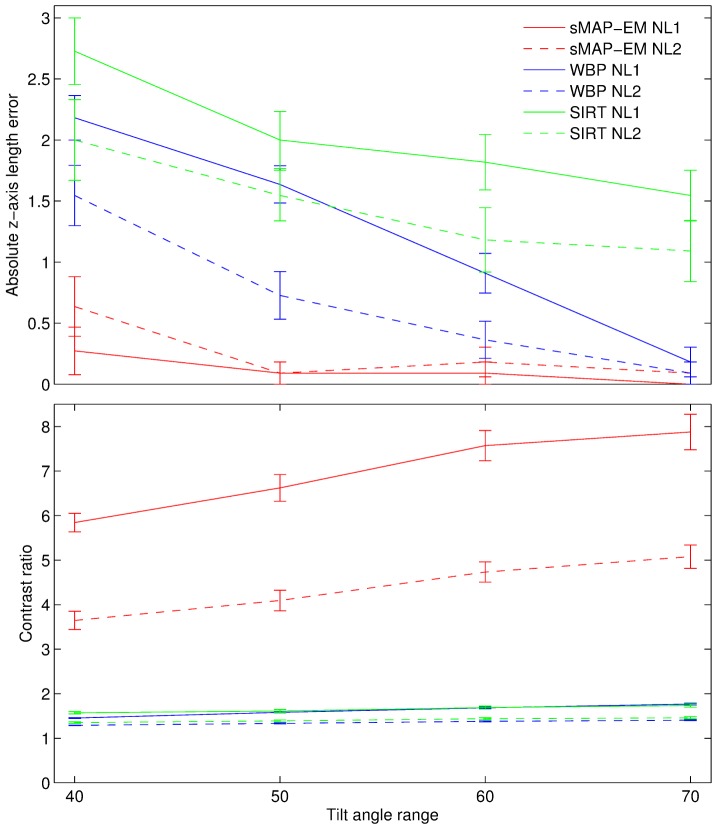
Effects of different missing wedge sizes to absolute z-axis length error and contrast ratio. Measured means and standard errors of the means of the absolute z-axis length errors and the contrast ratios of 11 gold particles in the cell phantom reconstructions. Measurements from the reconstructions of projections with noise level 1 (solid line) and noise level 2 (dashed line) are shown in the same plot. The tilt angle range is affecting the z-direction resolution with all methods. sMAP-EM is clearly the best and has the least effect of increased missing wedge. sMAP-EM gives the best contrast improving with the angular range while the WBP and SIRT reconstructions have low contrast with all tilt angle ranges.

**Figure 8:**
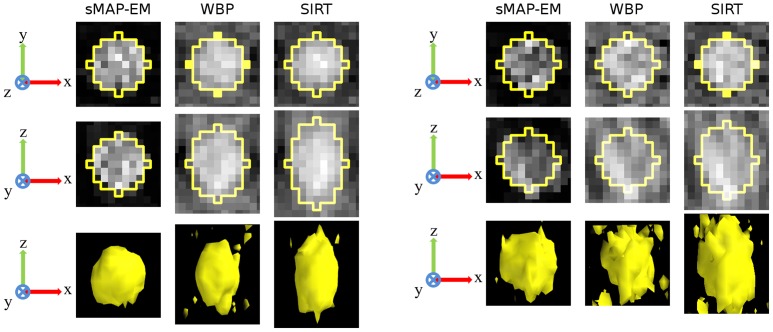
3D fitted ellipsoids on a representative gold particle in the cell phantom reconstructions. 2D orthogonal x–y (top) and x–z (middle) slices through the center of a 3D fitted ellipsoid drawn over a representative measured gold particle from reconstructions with projections in ±60° tilt angle range. Surface rendering of the gold particle (bottom) presents overall shape of the reconstructed gold particle. Isosurface threshold value was selected experimentally for the best visualization. All images are in the same scale. The full dynamic range of each image was used for visualization. Intensity inside the gold particle in the sMAP-EM reconstruction appears to have larger variation than in the WBP and SIRT reconstructions. This results partly from higher smoothing especially in the SIRT reconstruction, but also from the fact that the variation of the background intensity in the WBP and SIRT reconstructions is high and the contrast of the gold particle is low making the inside of the gold particle appear more uniform in the visualization. The contrast of the gold particle is high in the sMAP-EM reconstructions from (A) noise level 1 and (B) noise level 2 projections making fitting accurate. The contrast of the gold particle is much lower in the WBP and SIRT reconstructions especially with noise level 2 projections, reducing the accuracy of the ellipsoid fitting as presented quantifically in Figure S1.

The quantitative evaluations for the accuracy and the CR of 11 gold particles in the cell phantom reconstructions of both noise levels and all four tilt angle ranges (±40°, ±50°, ±60°, ±70°) are presented in Figure 7. The results of the ellipsoid fitting method (Figure 8) show that the sMAP-EM reconstruction results are consistently better with extremely good accuracy. The method produced reconstructions with low elongation in the lateral plane (Table 1). The WBP and the SIRT reconstructions show an increase in elongation in the z-direction, as measured by the fitted ellipsoids. The WBP reconstruction also results in elongated gold particle images in the lateral plane. From these results we can see that the sMAP-EM produces the best isotropic resolution in all directions that is close to the ground-truth value.

**Table 1:** Quantitative results for 11 gold particles in the cell phantom reconstruction.

Noise	Method	LAE*_x_*	LAE*_z_*	*e_yx_*	*e_zx_*	*e_zy_*	CR
NL 1	sMAP-EM	0.00±0.00	0.09±0.09	1.00±0.00	1.03±0.03	1.03±0.03	7.57±0.34
	WBP	0.36±0.15	0.91±0.16	1.11±0.05	1.36±0.08	1.23±0.05	1.68±0.02
	SIRT	0.09±0.09	1.82±0.23	1.03±0.03	1.50±0.05	1.46±0.05	1.69±0.04
NL 2	sMAP-EM	0.00±0.00	0.18±0.12	1.00±0.00	1.06±0.04	1.06±0.04	4.73±0.22
	WBP	0.45±0.16	0.36±0.15	1.15±0.06	1.27±0.11	1.09±0.04	1.38±0.01
	SIRT	0.09±0.09	1.18±0.26	1.03±0.03	1.32±0.06	1.29±0.06	1.44±0.02

Quantitative results of cell phantom reconstructions with ±60° tilt angle range. LAE*_x,z_* presents mean of fitted ellipsoid axis length absolute error in pixels, *e_yx,zx,zy_* is mean ellipsoid elongation and CR is mean contrast ratio. Standard error of the mean is included with the mean values. LAE*_y_* is excluded from the table because none of the methods show any error in fitted ellipsoid y-direction.

Contrast and the amount of noise in reconstruction is important for further automatic and semi-automatic analysis and visualization. The effects of different noise levels in projections was studied to see how much different methods are influenced by the noise. We used five different noise levels in projections in ±60° tilt angle range and measured gold particle elongation. With perfect reconstruction method the elongation should be independent of the noise and only dependent of the missing wedge. The results (Figure S1) show that gold particles are analysed reliably from sMAP-EM reconstructions of all noise level projections. The measured elongation increases slightly due to increased noise in the reconstructions. However, the contrast becomes very low especially with the largest noise level in the WBP and SIRT reconstruction preventing reliable automatic measurements of the gold particles seen as sudden drop of the elongation measurement. The CR results (Figure 7) show that the sMAP-EM reconstruction produces much better contrast than the WBP and the SIRT reconstructions. This is visible also in Figures 6 and 8.

**Figure 9:**
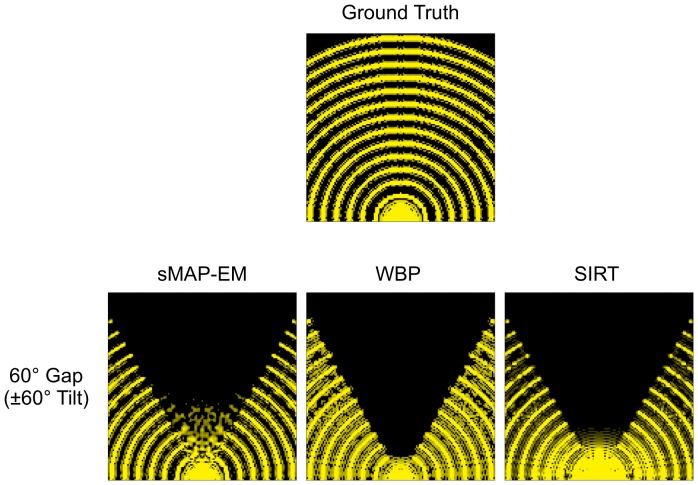
Observation of the missing wedge in the frequency domain. Along with the ground truth, the spectra of the synthetic pattern reconstructed by sMAP-EM, WBP, and SIRT are shown. The width of the missing wedge used is ±60°. For better visual comparison, all amplitudes having lower than a threshold value were set to zero and the spectrum was scaled with the logarithm of base 10; the threshold value and scaling factor were adjusted for each reconstruction method. The filling of the missing wedge by sMAP-EM with meaningful information is supported by quantitative results of gold particle studies presented in Figure 7 and Tables 1 and 2.

To observe how the missing wedge is filled by different reconstruction methods with different wedge sizes, we reconstructed the projections calculated from the synthetic slice shown in Figure 1D. The comparison of the resulting spectra shows that sMAP-EM filled more information in the missing region, while the SIRT and WBP failed to do so (Figure 9). The overall trend of the sMAP-EM reconstructions shows that recovery of the low frequency was more recognizable than that of the high frequency in the missing regions. Furthermore, the accuracy of the filling deteriorated as the wedge angle increased (Figures S2 and S3). The amplitude and connectivity of the bridging decreased with the increasing frequency. In the reconstruction with 60° wedge, the information fillings were recognizable at the edge of the region but were blurry in the middle if there were any, resulting in the disconnection of the bridging arcs. However, the other results like the elongation of the gold particles show that the filled information is useful.

### Experimental data reconstructions

**Figure 10:**
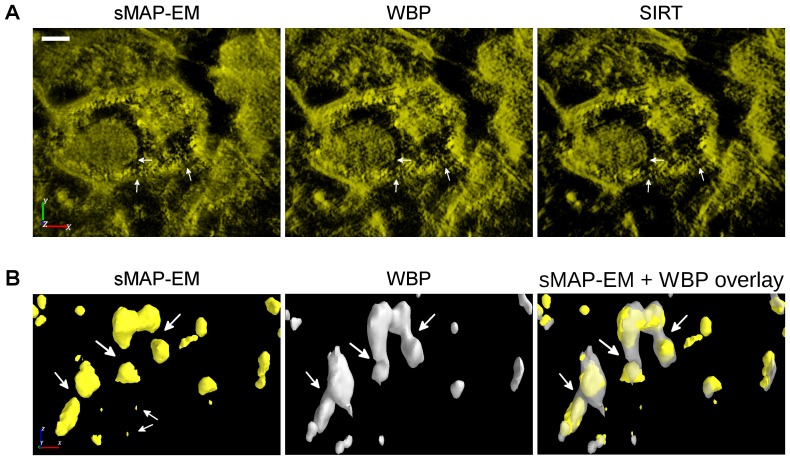
3D rendering of the experimental vesicle data reconstructions. Volume renderings (A) show that the MVB membrane and its breakages (marked with white arrows) are visible with all reconstruction methods. However, large intraluminal vesicle and its membrane (marked with an arrow) is much more clearly visible in the sMAP-EM reconstruction than in the WBP and SIRT reconstructions. The sMAP-EM reconstruction can be directly volume rendered without any thresholding whereas both WBP and SIRT need a threshold to remove the background to make interesting regions visible. The threshold selection is a qualitative process which can lead to the removal of interesting data while reducing the background. For this figure, the thresholds for WBP and SIRT were selected for the best possible presentation. Scale bar 100nm. MVB membrane structure was further studied with surface rendering (B) from the direction of y-axis. The sMAP-EM reconstruction was compared to WBP which was quantitatively 2 shown to produce less elongation than SIRT. It is clearly visible that sMAP-EM is able to distinguish individual particles (regions marked with arrows) where WBP creates elongated connection. Also small particles (marked with arrows) are visible in the sMAP-EM that are absent in the WBP. The isovalues for surface renderings were selected so that the same percentage of the highest density voxels were included in the surface with both methods.

3D rendering of the reconstructions (Figure 10) show clear differences between methods especially on the x–z direction where the missing wedge mostly effects. The membrane of the MVB is clearly distinguishable with membrane breakages in all volume renderings (Figure 10A). Membrane of the large intraluminal vesicle is more connected in the sMAP-EM reconstruction. When visualizing the MVB membrane particles from sideview (Figure 10B) the difference in elongation between sMAP-EM and other methods comes clear. sMAP-EM is able to separate particles in the membrane where WBP creates elongated connection. As a result of different background levels, artifacts and contrast, preparation of the visualization is different in sMAP-EM than in WBP and SIRT. The sMAP-EM reconstruction can be directly volume rendered without setting any threshold value whereas often complicated and possibly even biased use of manual thresholding to remove the background have to be done to visualize interesting structures from the WBP and SIRT reconstructions.

**Figure 11:**
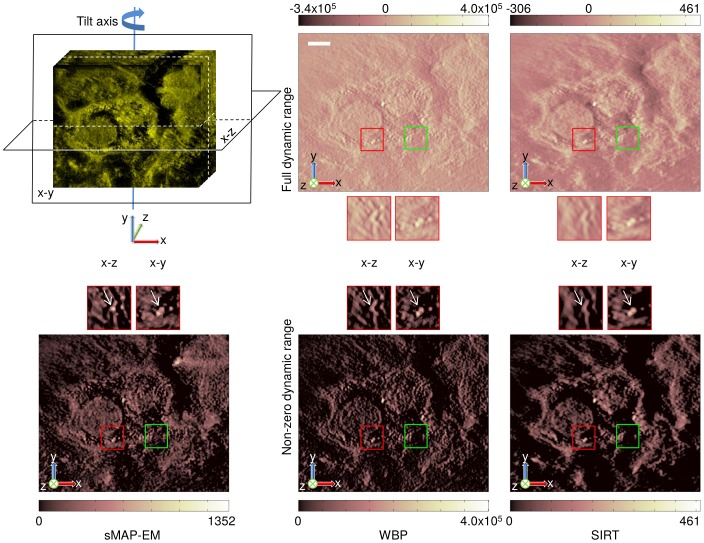
Comparison of orthogonal slices between sMAP-EM, WBP, and SIRT reconstructions from the experimental dataset. (Top) The leftmost figure presents the location of the orthogonal x–y and x–z slices over the reconstructed volume. Full dynamic range of each slice is visualized with a pseudo-color. Two boxes below each image are zoomed-up views of red region of interest (ROI) and its corresponding x-z plane. Green box in all slices indicate location of presumable MVB membrane breakage. (Bottom) Only non-negative pixel values of each slice are rendered with the same pseudo-color, by setting zero to all pixels originally having negative values. Two boxes above each image are zoomed-up views of the same ROI as in the images at the top, and associated x–z plane. sMAP-EM yields identical results for full and non-zero dynamic ranges while WBP and SIRT result in different visual impressions. sMAP-EM is superior to WBP and SIRT to reveal association of gold particles (white arrows). Scale bar 100nm.

**Figure 12:**
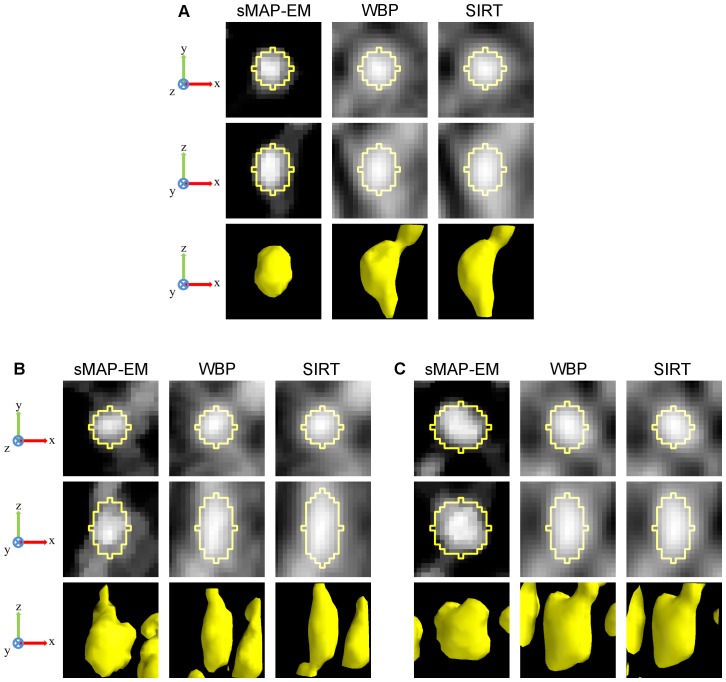
3D fitted ellipsoids on gold particles in the experimental vesicle data reconstructions. Subfigures A, B, and C present reconstructions for three different gold particles. For each gold particle, orthogonal x–y (top) and x–z (middle) slices through the center are given. Surface renderings (bottom) present overall shape of the reconstructed gold particles. Isosurface threshold value was selected experimentally for the best visualization. All images are in the same scale. The full dynamic range of each subimage was used for the best visualization. The z-direction resolution is better in the sMAP-EM reconstruction than in the WBP and SIRT reconstructions. Also the contrast is superior in the sMAP-EM reconstruction making further analysis simpler. The visual impression is supported by quantitative results presented in Table 2.

In Figure 11, orthogonal slices of the experimental data are presented first in the original scale and then as scaled to positive values to be comparable. Volumes of the sMAP-EM reconstruction are identical in both full and non-negative dynamic range since this method guarantees the value “zero” to be the baseline indicating no detectable densities. However, WBP and SIRT slices give considerably different visual impressions because the baseline values were not kept to be zero in addition to the negative value artifacts. Effects of these differences on visualization are clear in the zoomed ROIs. The gold particle in the red box is clearly visible in the sMAP-EM reconstruction but show elongation and less contrast in other reconstructions. The green box is located on presumable MVB membrane breakage. Furthermore, an isolated gold particle can be seen above the green box. Its intensity in the bottom visualization supports the superior contrast of sMAP-EM over SIRT and WBP. A bright object on the right-top side in the x-y slice of the sMAP-EM also shows the visibility difference; this was consistent in the z-direction neighbors of x-y slices. In general, the gold particles are much more distinguishable in the sMAP-EM reconstruction compared with the WBP and the SIRT reconstructions. The same can be seen in the ellipsoid fitting (Figure 12, Figure S4) on a gold particle.

The quantitative results of gold particle elongation (Table 2) show that sMAP-EM reduce elongation as compared to WBP and SIRT. This is important when studying connectivity of particles in the z-direction that is the most important direction gained from tomography studies. Gold particles for the ellipsoid fitting based evaluations were identified manually. 20 individual gold particles were detectable in sMAP-EM reconstruction. However, as a result of poor contrast and artifacts in the WBP and the SIRT reconstructions, only 7 isolated gold particles were found. These were used for the quantification in all methods. The results of the ellipsoid fitting based evaluations of isotropic resolution in the reconstructions are presented in Table 2. The sMAP-EM reconstruction was the most symmetric in x, y, and z-directions. The contrast ratio in the sMAP-EM reconstruction was almost three times higher than in other reconstructions. There is no elongation in the images of gold particles in the lateral plane in the SIRT reconstruction. However, the elongation in the z-direction in the SIRT reconstruction with long object compensation is larger than in the sMAP-EM and the WBP reconstructions. The WBP reconstruction shows elongation also in the lateral plane as a result of artifacts in x-direction.

**Table 2:** Quantitative results for 7 gold particles in the experimental vesicle data.

Method	*e_yx_*	*e_zx_*	*e_zy_*	CR
sMAP-EM	1.01±0.06	1.27±0.07	1.27±0.07	3.01±0.36
WBP	1.14±0.05	1.57±0.09	1.38±0.07	1.18±0.01
SIRT	1.00±0.00	1.67±0.14	1.67±0.14	1.18±0.01

Quantitative results of reconstructions of experimental data. Mean elongation (*e_yx,zx,zy_*) and mean contrast ratio (CR) of the gold particles are presented with standard error of the mean.

## Discussion

To use the advantages of MAP-EM for the missing wedge and the high noise level in input images, a modified version, “sMAP-EM”, was applied to the ET reconstruction of the biological samples in this work. Our results with both phantom and experimental datasets demonstrated that the sMAP-EM is consistently superior to the WBP and SIRT methods, in terms of the elongation suppression, 3D resolution, and contrast. The WBP and SIRT severely suffered from the missing wedge while the sMAP-EM handled this information loss better. This way sMAP-EM produced better isotropic resolution. Contrast between interesting and background regions in reconstructed volume determine how accurate automatic or semi-automatic analysis and visualization are. The sMAP-EM gave much better contrast than the WBP and SIRT in all experimental and simulated studies. This made quantitative analysis of gold particles (Figure S1) and visualization of experimental data reconstructions (Figure 10) more reliable without need for any user-defined parameters for sMAP-EM reconstructed volume.

The idea of sequential regularization is to fit a statistical model to the projection data while regularizing estimates sequentially. The weight of the regularization is kept high initially to strongly suppress any contamination and to get more robust estimates, and it is decreased sequentially to improve resolution of more reliable estimates. We assumed Poisson model for the projection data in the maximum likelihood estimation while penalizing the noise contamination with median filter regularization in this study with sMAP-EM. The results showed that the selected model and the regularization filter are quite suitable for reconstructing the ET data. However, different models and regularizations (e.g. L-filter [23]) can be used for the other tomographic imaging environments and target samples still taking advantage of sequential regularization. Use of *a priori* knowledge about the shape and density of the objects in the sample can further improve the results. Nevertheless, this data is not always available as we did not have it in our case.

The minimum required number of sequences and iterations for each sequence depend on variance of sample and noise level. It is known that high number of iterations generates checkerboard artifacts in the images reconstructed with statistical methods if the images are not regularized during the iterations [31]. However, sMAP-EM can use large number of iterations and keep improving the images since the weight of regularization is never set to zero. Therefore, the number of iterations was safely kept larger than sufficient for the datasets used to have a more general and robust sMAP-EM solution which can be applicable to various samples at different noise levels. The number of sequences and iterations for each sequence can be optimized using an adaptive strategy. Since there is no general image quality criteria to be used for this adaptation, the strategy should be determined according to the requirements of the specific application and the trade-off between the image resolution and the noise level. The high number of iterations increases the computation time. Nonetheless, it can be reduced easily by any computer grid since the 3D sample volume can be reconstructed slice by slice in parallel. We used Techila computer grid in this study to get the results within a feasible time. The demand for high resolution biological images is so high that long computation time can often be acceptable. Therefore, the sMAP-EM method could be a valuable method in cellular biology for further quantitative analysis of the ET reconstructions.

Interpolation [35] and image inpainting [36] methods can be used to recover the missing projection data. These methods use the data available around the edges of the information gap to fill the missing region. The accuracy of the filling highly depends on the size of the gap and the noise level of the projections. In ET, typically ±60° range is used to acquire projections and it is difficult for these methods to recover such a large gap for this range. sMAP-EM fills the gap in a different way by searching for the most likely image, given the whole available projection data. Using the whole available data in the maximum likelihood sense makes sMAP-EM a robust method for filling the missing wedge (Figure 9 and Figure S2). The missing information can also be reduced by using the double axis or the conical tilting geometry. Despite all the complications, if these techniques were used for data acquisition, multi-tilt extension of sMAP-EM would be the reconstruction method to be preferred. It is shown in Figure S3 that the gap filling strength and accuracy of sMAP-EM increase rapidly with the decrease of the missing information. Another observation for this figure is that the high frequencies are not recovered as well as the low frequencies in the missing wedge regions with different sizes. The loss of high frequency content in the missing wedge region decreases the uniformity of the resolution in the spatial domain. However, the other results indicate that even the weak gap filling for the ±60° tilt angle range is sufficient to produce significant elongation suppression, 3D resolution improvement, and contrast enhancement.

It is difficult to evaluate the accuracy of the experimental data reconstructions since the ground truth is not available. Even though, it is easier to make objective evaluations if some objects with known shape exist in the reconstructed volume. In this study, we had gold particles in the sample used for the experimental data, so we were able to use these particles as reference objects for the quantitative evaluations. For these evaluations, we developed an ellipsoid fitting based method enabling objective, parameter-free, and automatic analysis of the gold particles. In order to have global and statistical information about the reconstruction methods, several gold particles were selected randomly from various parts of the image volume for the evaluation. The method worked well in all sMAP-EM reconstructions. However, the lower contrast in the WBP and the SIRT reconstructions reduced detection accuracy, especially in the cell phantom experiment with the higher noise level (Figure 8, Figure S1).

Deducing the 3D accuracy was possible by comparisons between known radii of the gold particles and three semi-axis lengths of the corresponding fitted ellipsoids in the simulated cell phantom case. Along with our expectations, the results of the quantitative evaluations showed the sMAP-EM reconstruction to be much better than the WBP and SIRT with the test data, adding objective evidences to the visual inspections. The elongation measurements in the cell phantom experiment clearly demonstrated that the sMAP-EM reconstructions were virtually free from this artifact, while both WBP and SIRT reconstructions included the artifacts. The elongation was also consistently reduced in the experimental data reconstruction with the sMAP-EM method.

High contrast and resolution of the sMAP-EM method enables automatic analysis as well as better visual interpretation in ET imaging. For example, the experimental data in Figure 11 shows the localization of target proteins labelled with gold particles. The automation of location detection, counting, distribution calculation, and adjacent distance calculation of the gold particles in this dataset are easier with sMAP-EM.

The present research demonstrated that the proposed sMAP-EM method can successfully compensate for the missing wedge artifacts in ET while improving the contrast and the 3D resolution of the reconstructed structures from both simulated and experimental data. All the quantitative measures supported the qualitative evaluations and showed the sMAP-EM reconstruction to be consistently better than the WBP and SIRT. For accurate ET analysis of the complex cellular structures in biology, we believe that sMAP-EM is the method to be chosen.

## Supporting Information

Figure S1 **Effects of noise on projections to absolute z-axis length error in the sMAP-EM, WBP and SIRT reconstructions.** The plot presents absolute z-axis length error measured from the gold particles in the reconstructions of ±60° cell phantom projections with different noise levels. Higher noise level decreases the contrast of the reconstructions making the objective ellipsoid fitting less accurate. The noise levels 1 and 2 used in the study are 16.1% and 18.0%, respectively. The other tested noise levels have smaller noise contamination. An ideal reconstruction method independent of the noise in projections would result in straight line with zero slope. It is expected that when the noise level increases, the accuracy of automated analysis method decreases. The absolute z-axis length is slightly increasing with noise on sMAP-EM (red) reconstructions as expected. In the WBP (blue) and SIRT (green) reconstructions, the contrast becomes extremely low with the highest noise contamination that prevents reliable automatic analysis of the gold particles with the ellipsoid fitting method.
(EPS)

Figure S2 **Change in average amplitude ratio of missing per non-missing area relative to wedge size.** The graph shows that sMAP-EM fills information in the missing wedge with significantly larger average amplitude than WBP and SIRT. The difference is observed for all wedge sizes including the practical tilting angle ranges of electron tomography (gray-box). The decreasing trend of the ratios in all reconstruction methods is the same as expected; larger the missing area, less filling. However, the long object compensation of Tomo3D SIRT (green) suppresses this decreasing trend as compared to the SIRT without the compensation (cyan).
(TIF)

Figure S3 **Spectra of synthetic pattern sMAP-EM reconstructions with 10° increment step of the missing wedge size.** To reassure the deterioration trend of the gap filling accuracy relative to the increase of the missing information, sMAP-EM reconstructions with 10, 20, 30, 40, and 50° missing wedge were also conducted. The reconstruction of sMAP-EM with 60° missing wedge is compared to the WBP and SIRT reconstructions in the Figure 9 showing also the ground truth.
(TIF)

Figure S4 **The other 3D fitted ellipsoids in the experimental vesicle data reconstructions.** Orthogonal x-y (top) and x-z (middle) slices through the center of the gold particles. Surface rendering of the gold particle (bottom) presents overall shape of the reconstructed gold particle. Isosurface threshold value was selected experimentally for the best visualization. All images are in the same scale. The full dynamic range of each subimage was used for the best visualization. The quantitative results are presented in Table 2.
(TIF)
