# Supplementary figures and images for: Compensation of Missing Wedge Effects with Sequential Statistical Reconstruction in Electron Tomography
Source: PLoS One. 2014 Oct 3;9(10):e108978. doi: 10.1371/journal.pone.0108978 (PMC4184818; doi:10.1371/journal.pone.0108978)

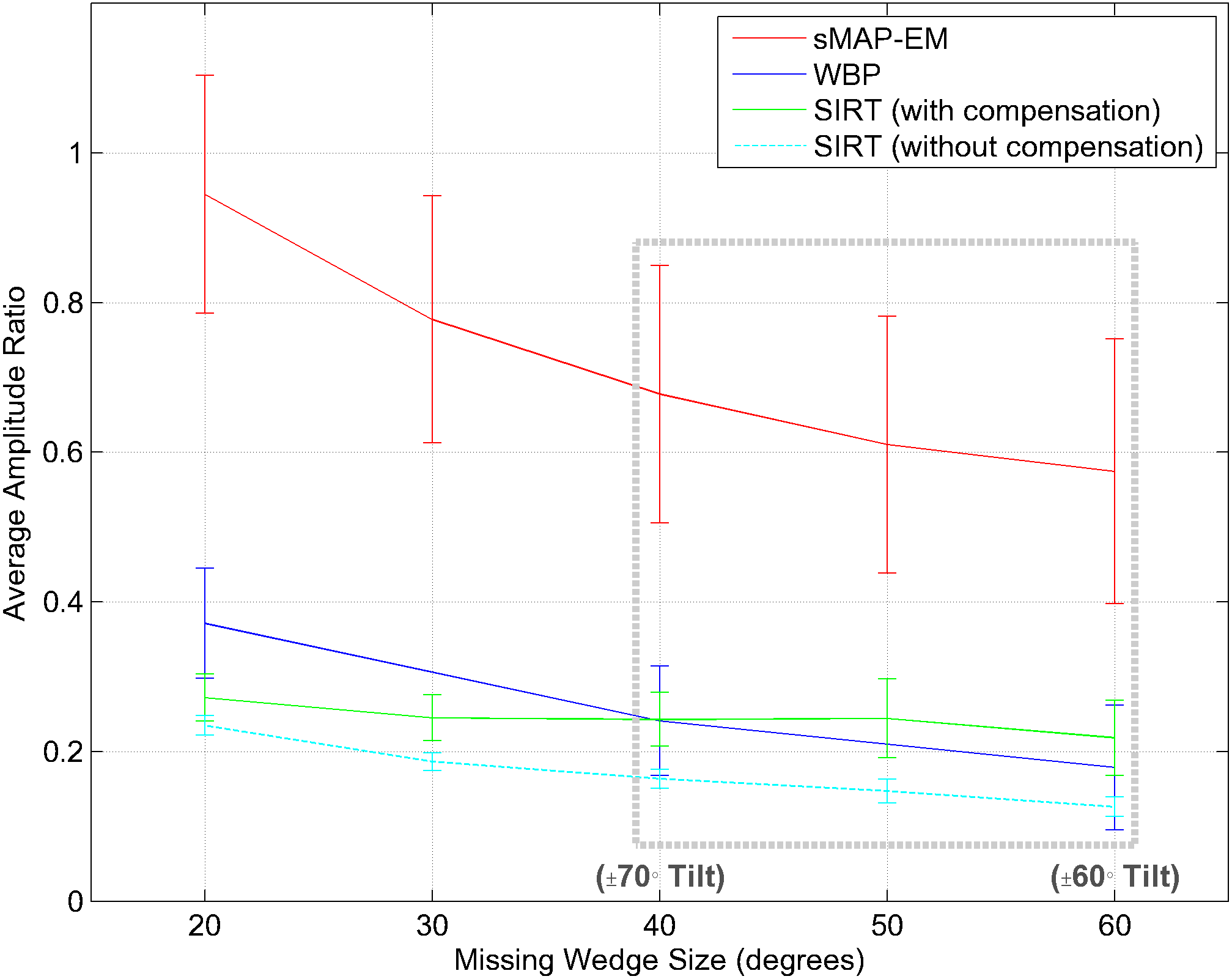

Supplement: Figure S2 — Change in average amplitude ratio of missing per non-missing area relative to wedge size. The graph shows that sMAP-EM fills information in the missing wedge with significantly larger average amplitude than WBP and SIRT. The difference is observed for all wedge sizes including the practical tilting angle ranges of electron tomography (gray-box). The decreasing trend of the ratios in all reconstruction methods is the same as expected; larger the missing area, less filling. However, the long object compensation of Tomo3D SIRT (green) suppresses this decreasing trend as compared to the SIRT without the compensation (cyan). (TIF) [file pone.0108978.s002.tif]

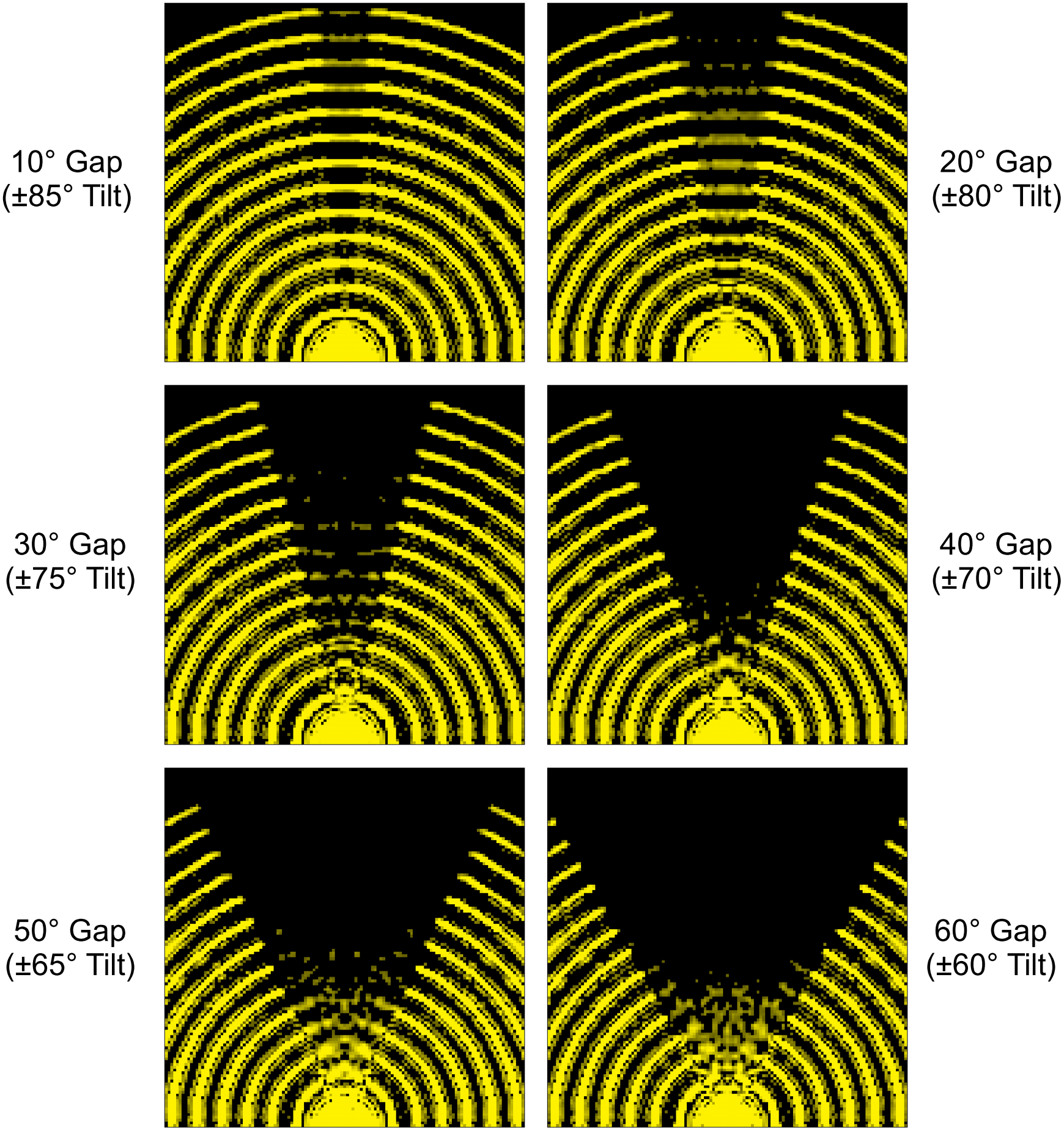

Supplement: Figure S3 — Spectra of synthetic pattern sMAP-EM reconstructions with 10° increment step of the missing wedge size. To reassure the deterioration trend of the gap filling accuracy relative to the increase of the missing information, sMAP-EM reconstructions with 10, 20, 30, 40, and 50° missing wedge were also conducted. The reconstruction of sMAP-EM with 60° missing wedge is compared to the WBP and SIRT reconstructions in the Figure 9 showing also the ground truth. (TIF) [file pone.0108978.s003.tif]

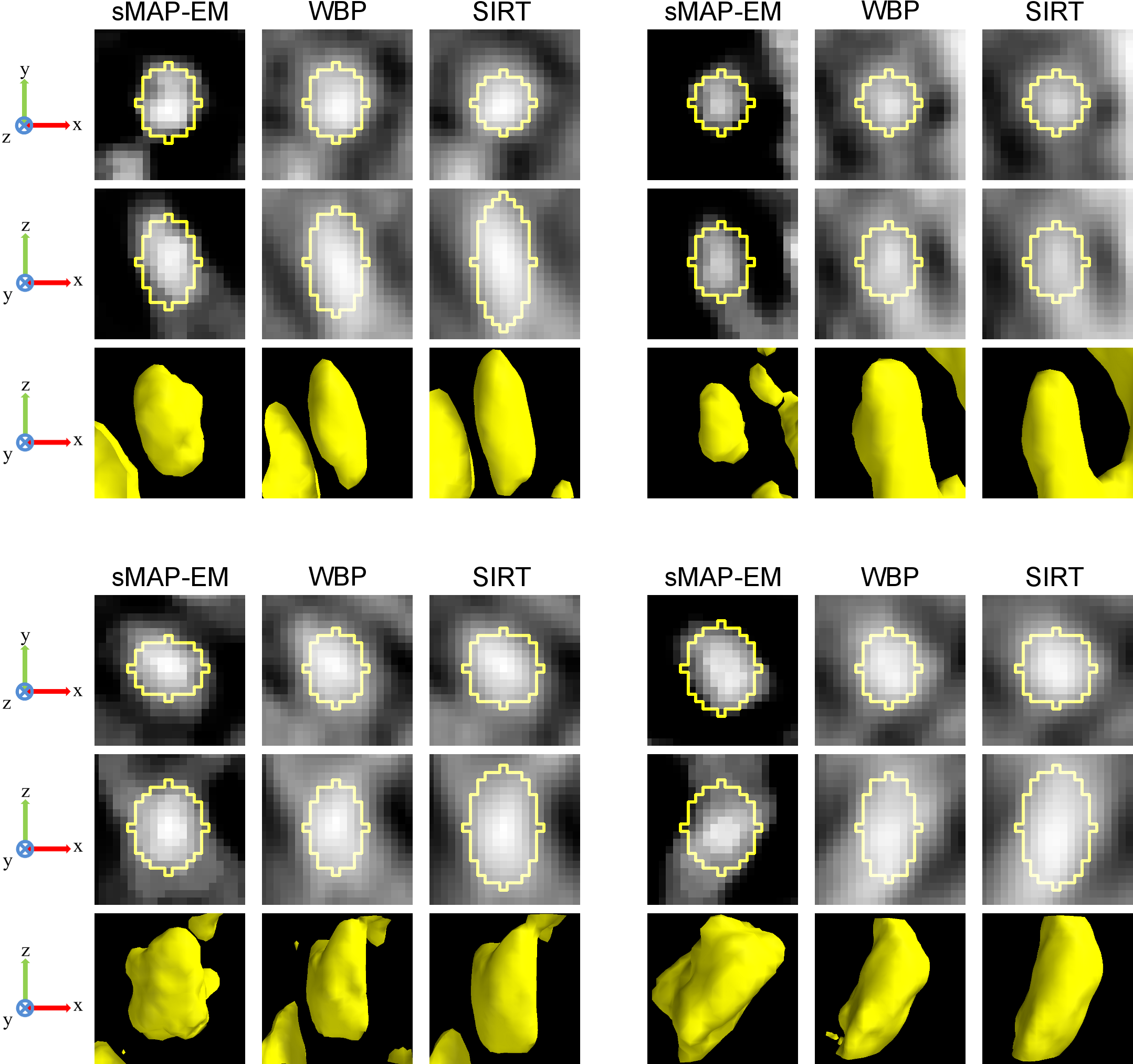

Supplement: Figure S4 — The other 3D fitted ellipsoids in the experimental vesicle data reconstructions. Orthogonal x-y (top) and x-z (middle) slices through the center of the gold particles. Surface rendering of the gold particle (bottom) presents overall shape of the reconstructed gold particle. Isosurface threshold value was selected experimentally for the best visualization. All images are in the same scale. The full dynamic range of each subimage was used for the best visualization. The quantitative results are presented in Table 2. (TIF) [file pone.0108978.s004.tif]
